# The Neonicotinoid Thiacloprid Interferes with the Development, Brain Antioxidants, and Neurochemistry of Chicken Embryos and Alters the Hatchling Behavior: Modulatory Potential of Phytochemicals

**DOI:** 10.3390/biology11010073

**Published:** 2022-01-04

**Authors:** Mayada R. Farag, Mahmoud Alagawany, Attia A. A. Moselhy, Enas N. Said, Tamer A. Ismail, Alessandro Di Cerbo, Nicola Pugliese, Mona M. Ahmed

**Affiliations:** 1 Forensic Medicine and Toxicology Department, Veterinary Medicine Faculty, Zagazig University, Zagazig 44519, Egypt; monaya@zu.edu.eg; 2 Poultry Department, Faculty of Agriculture, Zagazig University, Zagazig 44511, Egypt; 3 Department of Anatomy and Embryology, Faculty of Veterinary Medicine, Zagazig University, Zagazig 44519, Egypt; aameselhy@vet.zu.edu.eg; 4 Department of Veterinary Public Health, Faculty of Veterinary Medicine, Zagazig University, Zagazig 44519, Egypt; enmohamed@vet.zu.edu.eg; 5 Department of Clinical Laboratory Sciences, Turabah University College, Taif University, P.O. Box 11099, Taif 21944, Saudi Arabia; t.ismail@tu.edu.sa; 6 School of Biosciences and Veterinary Medicine, University of Camerino, 62024 Matelica, Italy; 7 Department of Veterinary Medicine, University of Bari ‘Aldo Moro’, 70010 Bari, Italy; nicola.pugliese@uniba.it

**Keywords:** chicken embryos, thiacloprid, chicoric acid, rosmarinic acid, developmental neurotoxicity

## Abstract

**Simple Summary:**

The present experiment was performed to investigate the toxic impact of thiacloprid (TH) on the brain of developing chicken embryos and also to measure its influence on the behavioral responses of hatchlings. The role of chicoric acid (CA) and rosmarinic acid (RA) in modulating the resulted effects was also investigated. TH resulted neurotoxic to chicken embryos and possibly neurotoxic to embryos of other vertebrates. Moreover, CA and RA exerted both an antioxidant and a neuroprotective effect on embryos.

**Abstract:**

The present experiment was performed to investigate the toxic impact of thiacloprid (TH) on the brain of developing chicken embryos and also to measure its influence on the behavioral responses of hatchlings. The role of chicoric acid (CA) and rosmarinic acid (RA) in modulating the resulted effects was also investigated. The chicken eggs were in ovo inoculated with TH at different doses (0.1, 1, 10, and 100 ug/egg). TH increased the mortality and abnormality rates and altered the neurochemical parameters of exposed embryos dose-dependently. TH also decreased the brain level of monoamines and amino acid neurotransmitters and decreased the activities of acetylcholine esterase (AchE) and Na^+^/K^+^-ATPase. The brain activity of catalase (CAT) and superoxide dismutase (SOD) was diminished with downregulation of their mRNA expressions in the brain tissue. When TH was co-administered with CA and RA, the toxic impacts of the insecticide were markedly attenuated, and they showed a complementary effect when used in combination. Taken together, these findings suggested that TH is neurotoxic to chicken embryos and is possibly neurotoxic to embryos of other vertebrates. The findings also demonstrated the antioxidant and neuroprotective effects of CA and RA. Based on the present findings, the CA and RA can be used as invaluable ameliorative of TH-induced toxicity.

## 1. Introduction

Neonicotinoids (neonics) are synthetic insecticides that have been extensively utilized for pest management and crop protection in domestic, agricultural, and veterinary practices since their introduction in the 1990s [1,2,3]. Neonics irreversibly bind to nicotinic acetylcholine receptors of target insects disrupting the transduction of neuron cholinergic signals and causing behavior abnormalities, immobility, and finally death of the insect [4].

Recently, it was reported that the widespread use of neonics may pose a potential risk to mammalian species, including humans, considering their capability of modulating the nicotinic receptors of non-target organisms and vertebrates [5]. 

Thiacloprid (TH) (Calypso^®^ 480 SC (480 g/L)) is a member of the neonics, which is still widely employed for the protection of vegetables, fruits, and ornamental plants species against voracious and sucking insects. It has been reported to cause hepatotoxicity [6], nephrotoxicity [7], and prostate toxicity [8]. Additionally, carcinogenicity, teratogenicity, other than fetal resorption, skeletal malformations in rat offspring, and altered motor and locomotor activities of adult rats have been observed [8]. Thiacloprid also altered the quality of chicken embryos [9] and preimplantation embryos of mice and rabbits [10]. Many natural compounds showed neuroprotective and antitoxic effects including, but not limited to, chicoric acid and rosmarinic acid [11,12,13].

Chicoric acid (CA) is a natural dicaffeyltartaric present in a wide variety of edible vegetables and plants such as the Mediterranean vegetables *Cichorium intybus* L. (chicory) [14], *Echinacea purpurea* [15], *Lactuca sativa* (iceberg lettuce) [16], *Taraxacum* spp. (dandelion) [17], *Ocimum* *basilicum* (basil) [18], some seagrass such as *Cymodocea nodosa* [19], and other plants. As a natural phenolic compound, CA was shown to possess anti-obesity, antioxidant, antiviral, anti-inflammatory, and immunomodulatory properties [20]. In addition, CA was shown to cross the blood–brain barrier providing strong free radicals scavenging and neuroprotective capacities [21]. Chicoric acid was found to suppress the inflammatory responses and improve the cell viability via redox-sensitive signaling pathways in BV2 microglial cells exposed to lipopolysaccharide [22] and in HepG2 cells after glucosamine induction [23]. It could also alleviate amyloidogenesis and cognitive impairments, suppress glial activation, prevent neuron damages, and decrease the inflammatory events in the central nervous system (CNS) [22,24]. 

Rosmarinic acid (RA) is also a natural hydroxylated polyphenolic substance found in many plant species like the *Lamiaceae* herbs *Rosmarinus officinalis* (rosemary), *Perilla frutescens* (perilla), *Melissa officinalis* (lemon balm), and *Onicum basilicum* (sweet basil). It is also present in *Blechnaceae* (a fern family)*, Boraginaceae*, and the seagrass family *Zosteraceae* [25,26]. Rosmarinic acid has been reported to exhibit anti-inflammatory, antioxidant, and antiviral actions in addition to angiogenic, hepatoprotective, anticancer, anti-neurodegenerative, and antidepressant properties [7]. 

In the light of the aforementioned considerations, this study aimed at investigating the toxic potential of TH on the developing brain of chicken embryos and its consequences on the behavior of hatchlings. In addition, the involvement of RA and CA in attenuating the TH-induced developmental neurotoxicity was elucidated. 

## 2. Materials and Methods

### 2.1. Chemicals 

**Figure 1:**
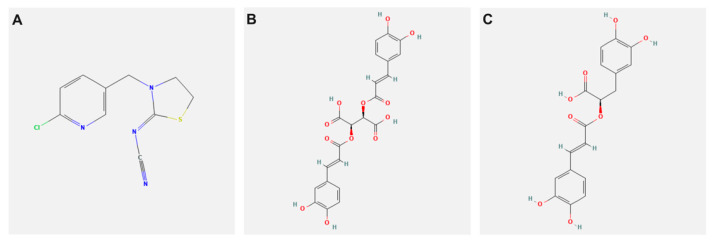
2D chemical structure representation of (**A**) Thiacloprid, (**B**) Chicoric, and (**C**) Rosmarinic acid (PubChem source).

Thiacloprid (TH) PESTANAL^®^, analytical standard, [C_10_H_9_ClN_4_S; [3-(6-Chloro-3-pyridinylmethyl)-2-thiazolidinylidene]cyanamide, with a molecular weight of 252.7 g/mol, CAS Number: 111988-49-9], Rosmarinic acid [purity ≥ 98%, C_18_H_16_O_8_, (R)-O-(3,4-Dihydroxycinnamoyl)-3-(3,4-dihydroxyphenyl)lactic acid, 3,4-Dihydroxycinnamic acid (R)-1-carboxy-2-(3,4-dihydroxyphenyl)ethyl ester, with a molecular weight 360.31 g/mol, CAS Number: 20283-92-5], and CA [purity ≥ 95%, C_22_H_18_O_12_ (2R,3R)-2,3-Bis{[(2E)-3-(3,4-dihydroxyphenyl)-1-oxo-2-propen-1-yl]oxy}butanedioic acid, 2,3-Di-trans-caffeoyltartaric acid, with a molecular weight of 474.37 g/mol, CAS Number: 70831-56-0], were purchased from Sigma-Aldrich International GmbH (St. Louis, MO, USA). The chemical structures of the tested compounds are presented in Figure 1. 

### 2.2. Eggs 

Fertilized chicken (Ross) eggs (weighing 60 ± 5 g) were obtained from a commercial hatchery. A total of approximately 780 eggs were utilized in 2 independent experiments. Proper management and care were provided for the hatching eggs and the chicks during the experiment. 

### 2.3. Experimental Design

#### 2.3.1. Experiment 1: Dose-Response

##### Management of Fertilized Egg

The egg surfaces were thoroughly cleaned with a solution of povidone-iodine and dried with clean dry tissue papers. After that, eggs were exposed to candling in a dark room to discard defective and broken eggs and to outline the air cell’s exact location using a pencil. A total of 360 fertilized eggs were included in this experiment and randomly allocated into six equal groups (60 eggs/group), eggs in each group were distributed into three subgroups, considered in the data analysis as independent replicates of each. Each egg of groups TH_0.1_, TH_1_, TH_10,_ and TH_100_ was treated with 0.1, 1, 10, and 100 µg TH, respectively, in 50 µL of sterile physiologic saline (the vehicle). The dose interval was selected to cover a wide range of environmental exposure according to previous observations [9,10]. Another group of eggs was inoculated with equivalent volumes of the vehicle. To evaluate the effect of the vehicle, a non-injected group (control) was used. Then the eggs were incubated at humidity 55%, temperature 37.8 °C, and turned once per hour. 

##### Air Cell Injections

On the third day of incubation, eggs were inoculated with the appropriate doses under sterile conditions. At the beginning of the embryogenesis process, TH was inoculated to allow its distribution within the entire organism, including the brain. The broad ends of the eggs were cleansed with a sterile gauze pad dipped in 70% ethanol. A hole was drilled by using a sterile needle in the shell over the air cell; care was paid to avoid damaging the shell membranes with the point of the drill. The needle was inserted horizontally into the air cell. After each injection, the needle was cleansed by means of a sterile gauze pad, and the shell holes were sealed with paraffin. 

##### Chicken Embryo Incubation

After inoculation of eggs, they were kept with air sac up with holes to permit airflow around them. Then the eggs were incubated at a 55% humidity, a temperature of 37.8 °C, and turned once per hour. The eggs were candled once a day to detect undeveloped and nonviable embryos, which were regularly recorded and removed.

On the 19th incubation day, eggs were opened, embryos carefully removed, separated from the yolk sacs, and washed with phosphate buffer saline (PBS). The rate of gross abnormalities, yolk-free body weight (YFBW), and mortalities was recorded. The brain was immediately removed and weighed. Brain samples were stored at −80 °C until neurochemical analysis. 

##### Evaluation of Brain Monoamines and Acetylcholine Esterase Activity

The levels of the monoamines dopamine (DA) and serotonin (5-HT) were estimated in brain homogenate by reverse-phase high-performance liquid chromatography equipped with an electrochemical detector (HPLC-ECD) using a C-18 column. Methanol/PBS (3:97, *v/v*) served as the mobile phase with a 1 mL/min flow rate as previously described [3]. 

The acetylcholine esterase (AChE) activity was evaluated according to the method proposed by Ellman et al. [27] by using a spectrophotometer at 412 nm. 

#### 2.3.2. Experiment 2: Antidotal Study

##### Experiment Design

Four hundred and twenty eggs were randomly divided into 7 groups, each divided into 3 groups of 20 eggs to allow 3 replications of the experiment. Thiacloprid (1 µg/egg), RA (100 µg/egg), CA (100 µg/egg) (each in 50 µL of sterile physiologic saline), and combinations of TH/CA, TH/RA, TH/CA+RA were administered to each group, respectively. Eggs of a control group were not inoculated. The doses of CA and RA were selected according to a previous study [2]. Fertilized egg management, air cell injection, chicken embryo incubation, collection of embryos (5/replicate), and brain sampling were performed as above described. The rest of the eggs were transferred to the hatchery and were placed in certain hatching boxes to perform behavioral observations on the hatchlings. 

##### Antioxidant Status in the Brain Tissue of Embryos

The brain specimens were homogenized in cold PBS, pH 7.5, in 1:5 *w*/*v* ratio using a Teflon homogenizer in an ice-cold water bath followed by centrifugation at 11.200 rcf at 0–4 °C for 15 min. The supernatant was collected and used to estimate the activity of superoxide dismutase (SOD) and catalase (CAT), and the content of reduced glutathione (GSH) by the colorimetric method as previously described [28,29,30]. 

##### Determination of the Activities of Acetyclcholine Esterase and Na^+^/K^+^-ATPase in Brain of Embryos

The activity of AChE was measured spectrophotometrically at 412 nm as previously described [27]. The activity of Na^+^/K^+^-ATPase was evaluated according to the method described by Agrahari and Gopal [31]. 

##### Determination of Brain Levels of GABA, Monoamines and Amino Acids of Embryos

The levels of γ-aminobutyric acid (GABA), 5-HT, and DA were evaluated in brain homogenates as mentioned in experiment one. 

The amino acid neurotransmitters aspartate, glutamine, and glycine were quantitatively estimated by fluorescence detection of *o*-phthaladehyde/2-mercaptoethanol (OPA/MCE) derivatives of the analytes, then separated by a gradient elution reversed-phase HPLC (Pre-column derivatization and reversed-phase HPLC) [32]. 

##### Transcriptional Analysis of Antioxidant-Related Genes in the Brain Tissue of Embryos

Total RNA extraction using TRIzol reagent (easyREDTM, iNtRON Biotechnology, Korea) was conducted on frozen brain samples. The synthesis of the first strand of cDNA from the extracted RNA was achieved by the mean of the Quantitect^®^ Reverse Transcription kit (Qiagen, Germany) following the manufacturer’s instructions. The forward and reverse sequences of primers of the investigated genes, namely, Superoxide dismutase 1 (SOD1), CAT, the Glutathione S-transferase class-alpha (GST-α), and the housekeeping gene β-actin, are listed in Table 1. 

T cDNA was used for qPCR analysis, carried out by using the QuantiTect^®^ SYBR^®^ Green PCR kit (Qiagen, Germany) in a Rotor-Gene Q instrument. The thermal cycle was as follows: 10 min at 95 °C, followed by 40 cycles of 95 °C for 15 s and 60 °C for 30 s, and 72 °C for 30 s. A melt-curve analysis was performed to verify the specificity of PCR. The relative mRNA expression pattern for each gene was calculated using the comparative 2^−ΔΔCt^ method [33].

### 2.4. Behavioral Responses of Hatchlings

To analyze motor activity and coordination, 3 behavioral categories were investigated, specifically the righting response, level balance beam, and startle response. From 2 to 7 days post-hatch (PHD), in the morning, the chicks were placed in 40 cm × 60 cm × 50 cm wooden boxes and their behavior was observed after 5 min acclimation. Specifically, the behavior of each chick was video-recorded for 10 min. Behavioral analyses were not carried out on PHD 1 because chicks of both control and treated groups moved very little or refuse to move. 

#### 2.4.1. Righting Response Test

Righting response test was performed to assess the vestibular functions and basic motor coordination [34]. The ability of chicks to right themselves was assessed by placing them on their back and by recording the time required to return to the right position. The tested chick was allowed to remain on its back for a maximum of 2 min. 

#### 2.4.2. Level Balance Beam

Level balance beam was used to reproduce novel situations, such as depth and height, to the newly hatched chicks and to assess fear-related behaviors (such as height fear) and the respective avoidance responses. The chicks were placed on a 6 cm × 35 cm level balance beam elevated over soft bedding by 22 cm to avoid chicks injuring when jumping [34]. Movement of chicks, latency (the time taken by chicks before jumping), and the distance walked by chicks on the balance beam were recorded. The test lasted for a maximum of 2 min. 

#### 2.4.3. Startle Response

Startle response was used to assess the auditory habituation according to Rogers et al. [35]. Chicks were observed in pairs after being placed in paper-lined wooden boxes (20 cm × 25 cm × 20 cm) and allowed to peck and move freely. Two polypropylene blocks were slammed one against each other every 30 s and the chicks were observed for the startle responses, e.g., head lift, flinching, or disruption from their activity. When a chick did not respond for 2 consecutive slams, the test was terminated and the number of the last startle responses was recorded as the score.

### 2.5. Data Analysis

Data were analyzed by using one way-ANOVA. Duncan’s Multiple Range test was performed to compare mean values between groups. Data were expressed as mean ± SEM. A value of *p* < 0.05 was considered statistically significant.

## 3. Results

### 3.1. Experiment 1: (Dose-Response Study)

#### 3.1.1. Effect on Mortality and Macroscopic Examination

Data in Table 2 showed no significant differences in the mortality rate of control, vehicle, and TH_0.1_ groups. 

Consistently, no macroscopic malformations were observed in those groups. Both mortality and abnormality rates were significantly higher in groups (*p* < 0.001) treated with higher doses of TH with respect to vehicle, and control groups. The effects were found to be more prominent in TH_100_, in which lack of reabsorption of the yolk sac, beak not formed, and incomplete formation of abdomen and organs were observed, followed by TH_10_ and then TH_1_ group. 

No significant changes in the relative brain weight or YFBW among control, vehicle, and TH_0.1_ groups were recorded, but they showed a significant decrease with respect to the vehicle and control groups following exposure to higher doses of TH (*p* < 0.001). The reduction was more prominent when doses increased from 1 to 100 µg/egg. 

#### 3.1.2. Effect of Thiacloprid on Acetylcholine Esterase and Monoamine Neurotransmitters

TH at 0.1 µg did not affect neurochemical parameters, except for AChE activity, which decreased. Conversely, the other TH-treated groups exhibited a significant (*p* < 0.001) decrease in the AChE activity and DA and 5-HT concentration. The effect of TH on AChE and monoamine neurotransmitters was found to be dose-dependent.

Given that the TH 1 µg/egg was found to be the minimal dose able to affect embryos, therefore, it was chosen to be used in the second experiment. 

Since no significant differences in mortalities or malformations occurrence were observed between the vehicle and the control groups, the vehicle group was considered as the control one in the second experiment. 

### 3.2. Experiment 2: Antidotal Therapy Study

#### 3.2.1. Effects on Antioxidant Variables

The SOD and CAT activities were lowered significantly (*p* < 0.001) in the brain tissues of TH-treated embryos if compared to the control. Upon separate co-administration of CA or RA with TH, a significant improvement of the inhibited activity of such enzymes was recorded (Table 3). 

The combined administration of CA and RA showed a complementary effect in restoring enzymes activities up to the level of the control group. Additionally, while TH induced a significant (*p* < 0.001) decline in the GSH level in the brain of treated embryos, separate or combined exposure to CA and RA restored the GSH level. When not administered concurrently to TH, CA, or RA did not significantly change SOD and CAT activities nor the GSH level (Table 3).

#### 3.2.2. Effects on AChE and Na^+^/K^+^-ATPase Enzymes Activity in the Brain

The activity of Na^+^/K^+^-ATPase and AChE was significantly (*p* < 0.001) reduced in the TH-treated group. Upon the CA and/or RA co-administration with TH, an improvement in the activity of AChE was recorded, particularly in the TH/CA+RA group, despite the overall activity remaining significantly lower than control (*p* < 0.001). 

Although the activity of the Na^+^/K^+^-ATPase was found reduced after treatment with TH, it was not found significantly different in TH/CA+RA group, compared to the control one (Table 3).

#### 3.2.3. Effects on the Brain Levels of GABA, Monoamines, and Amino Acids

The concentration of DA, 5-HT, GABA, and amino acid neurotransmitters in the brain of treated and untreated chicken embryos are reported in Table 3. A significant decrease in monoamines concentration was reported in TH-exposed embryos. A significant (*p* < 0.001) improvement was recorded when eggs were also inoculated with CA and RA, separately or in combination. Complete recovery of monoamine concentration was observed in the TH/CA+RA group, while GABA level was equally improved by separate or combined administration of CA and RA with TH (*p* < 0.001). 

There was a significant decrease in the glycine, aspartic acid, and glutamic acid level in the brain of TH-exposed embryos compared to the control. Separated or combined administration of CA and RA with TH significantly increased the concentration of the two acids to values comparable with those of the control group (*p* < 0.05). 

#### 3.2.4. Transcriptional Profile Changes in Antioxidant-Related Genes

The exposure to TH elicited significant downregulation of the SOD1 and CAT expression pattern compared to the control without any significant effect on GST-α mRNA expression. 

**Figure 2:**
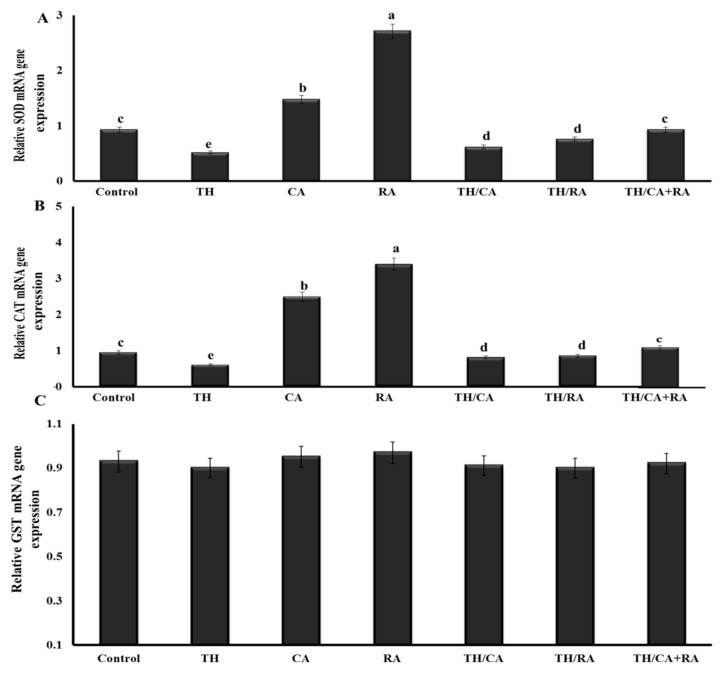
Effect of thiacloprid (TH), chicoric (CA), and rosmarinic acids (RA) in ovo exposure on the expression pattern of antioxidant-related genes (**A**) SOD, (**B**) CAT, and (**C**) GST-α in the brain of 19-day-old chicken embryos. Values are mean ± SEM, bars that are not sharing a common superscript letter (a–d) differ significantly at *p* < 0.05.

The downregulation was less evident when CA and/or RA were co-administered with TH, up to values comparable to control in the TH/CA+RA group (Figure 2). 

#### 3.2.5. Behavioral Response 

##### Effects on the Vestibular and Motor-Related Righting Response

**Figure 3:**
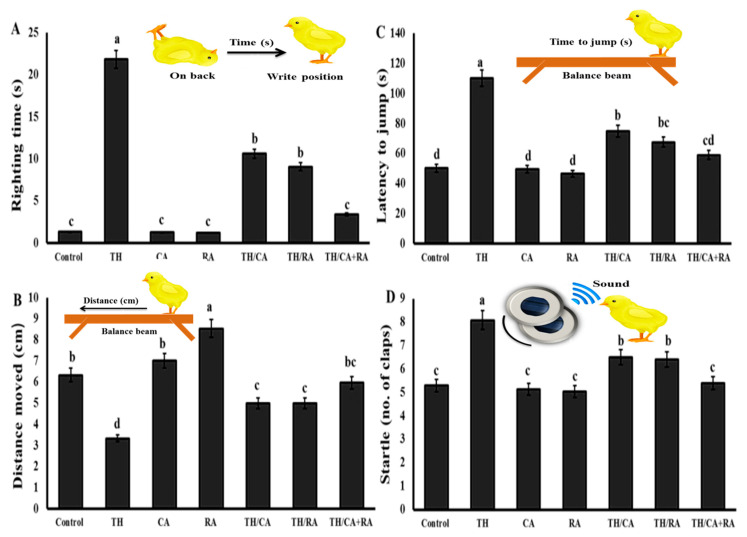
Effect of thiacloprid (TH), chicoric (CA), and rosmarinic acids (RA) in ovo exposure on (**A**) Righting time, (**B**) Distance moved, (**C**) Latency to jump, and (**D**) startle. Values are mean ± SEM, bars that are not sharing a common superscript letter (a–d) differ significantly at *p* < 0.05.

The mean righting time was longer in the TH group if compared to the control group. A significant reduction in the righting time was observed in the groups co-exposed to TH, CA, and/or RA. The righting response did not significantly change among the hatchlings belonging to CA, RA, and TH/CA+RA group when compared to the control group (Figure 3A).

##### Effects on Fear-Related Avoidance Behaviors

The shortest and the longest distance moved by chicks were recorded in the TH and RA groups, respectively (Figure 3B). 

The distances moved by chicks of the CA group were similar to the control. The chicks from the CA- and/or RA-co-exposed groups co-exposed to TH and CA and/or RA show an improvement in motility if compared to those from the TH group, mainly in the TH/CA+RA group (*p* < 0.05).

Exposure to TH during the embryonic stage significantly increased the fear and avoidance response, represented by the increase in latency to jump relative to control. The latency to jump did not significantly differ in CA, RA, and control chicks, while it significantly (*p* < 0.001) declined in TH/CA and TH/RA, and was up to comparable to the control in TH/CA+RA group (Figure 3C).

##### Effects on Auditory Habituation (Startle Response)

Figure 3D shows that the number of slams necessary for preventing chicks startling did not significantly differ among newly hatched chicks of CA, RA, and TH/CA+RA groups when compared to the control group (*p* < 0.05). Conversely, chicks from the TH group required a significantly higher number of slams than control and other groups (*p* < 0.001). Chicks from TH/CA and TH/RA groups showed a significant decline (*p* < 0.001) in the numbers of slams when compared to the TH group, despite remaining still higher than control.

## 4. Discussion

This experiment showed that the exposure to 1 µg/egg TH led to a reduction in embryo viability, an increase in abnormality risk, a decrease in brain weight and YFBW, and an alteration of brain neurochemistry. The quantity and severity of effects were found to increase with the concentration, suggesting a dose-dependent fashion. Similarly, TH induced developmental alterations and growth impairments, defects of limbs, and ectopia in chicken embryos at very low concentrations even lower than 1 µg/egg [9], where they used the minimum dose recommended by the pesticide data sheet, diluted in scalar proportions.

The potential of neonicotinoids to induce morphological abnormalities has been previously reported [36,37]. 

Additionally, interferences of imidacloprid with neuromuscular nicotinoid acetylcholine receptors have been recognized as possible causes for embryonic joint curvatures [38]. Babeľová et al. showed that pure and commercial products of TH, thiamethoxam, acetamiprid, and clothianidin negatively affected the quality and developmental potential of pre-implantation embryos of mouse and rabbit, particularly at 100 μM, the highest tested concentration [10]. However, these effects were absent at concentrations below 10 μM. They concluded that TH showed embryotoxicity, impaired quality, and development, and increased in the incidence of cell death of both rabbit and mouse pre-implantation embryos even at acute reference dose (daily exposure without deleterious effect; 0.03 mg/kg). 

Gu et al. reported that imidacloprid and acetamiprid at 500 μM markedly reduced the rate of morulae/blastocyst formation in culture from zygotes of naturally fertilized mice, while no effects were observed on the developmental ability of embryos cultured from the two-cell stage [39]. This may indicate that the sensitivity of embryos may differ according to the neonicotinoid compounds as previously observed [10]. The embryonic cells are well known for their high sensitivity to oxidative damage and inability to get rid of damaged cells by the apoptotic process [40]. This may explain the resulting negative effects of TH even at a dosage of 1 µg/egg. However, the underlying mechanism by which TH produces its developmental toxicity is still unidentified.

This study demonstrated that CAT and SOD activities in the brain, along with GSH content, were significantly lowered in TH-treated embryos in addition to downregulation of the SOD1 and CAT expression pattern. These situations may induce impairments in the antioxidant mechanisms and metabolic detoxification of embryos. Oxidative damage of TH was previously observed in the brain of chicken embryos [2]. 

The present results also agree with those by Aydin, who found that oral administration of TH decreased the level of antioxidants, antioxidant enzymes, and glutathione in lymphoid organs and the plasma of rats [41]. Similarly, reduced SOD, CAT, and glutathione peroxidase levels after TH exposure were reported by Feng et al. [42]. Kapoor et al. reported similar findings in the livers, kidneys, and brains of rats after administration of imidacloprid [43]. 

Other neonicotinoids, such as imidacloprid [44,45], thiamethoxam, and nitenpyram [46], changed the antioxidant biomarkers of fishes. The significant reduction in the activity of SOD observed in the TH group could be probably attributed to the excessive ROS production and the inability of the antioxidant system to thoroughly remove the excess free radicals from the body [47,48,49]. Neonicotinoids have also been reported to attack mitochondria, impairing their functions and increasing ROS production [50]. 

The enhancement of the activity of antioxidant enzymes acts as a buffer in the interception and detoxification of ROS and other various free radicals. In the current study, CA and RA markedly offset the oxidative injury provoked by TH with the recovery of CAT and SOD activities and expression of CAT and SOD1 gene, demonstrating mitigation of the oxidative burden. 

However, the protective effect was more prominent in the embryos co-exposed to both acids, indicating the presence of a complementary effect. 

Chicoric acid had been found to reduce the oxidative damage induced by TH in the brain of chicken embryos [2]. It increased the liver activities of antioxidant enzymes in mice [51], and CA supplementation ameliorated cognitive impairment (learning and memory loss) induced by D-galactose and prevented the H_2_O_2_-induced apoptosis of SH-SY5Y cells by promoting the Keap1/Nrf2 signal pathway and its downstream antioxidants in the brain of mice [24]. 

The regulation mechanism of CA may balance the cellular redox status, reverse the dysfunctions of mitochondria, decrease neuron apoptosis and inflammation induced by oxidative damage [20]. Several studies about the distribution and metabolism of CA indicated that it can penetrate the blood–brain barrier and exhibit free radical scavenging capacities, indicating that CA is a potential enhancer of the brain’s antioxidant activities [21]. CA was reported to suppress the inflammatory responses in BV2 microglial cells after exposure to lipopolysaccharide [22] and in HepG2 cells exposed to glucosamine [23] and enhance their viabilities via redox-sensitive signaling pathways. This experiment confirms previous results and provides scientific bases for the application of CA to mitigate the potential adverse effects of neonicotinoids, other than as a natural antioxidant and nutrient for the developing embryo.

Rosmarinic acid has been reported to exhibit anti-inflammatory and antioxidant effects properties [7]. Additionally, RA was proved to stimulate the antioxidant enzyme heme oxygenase-1 (HO-1) in association with the signaling pathways of phosphatidylinositol-3-kinase (PI3K) and the protein kinase A (PKA) [52]. Rosmarinic acid also showed neuroprotective activity against oxidative injury in the kindling epilepsy models [53]. 

Essential oils from RA prevented the liver damages induced by carbon tetrachloride in rats by normalizing the antioxidant activity of catalase, glutathione peroxidase, and glutathione reductase [54]. It was also found to reduce the TH-induced oxidative damage in the brain of chicken embryos [2]. These activities of both acids clarified their complementary effect in reducing the negative impact of TH. 

In the present study, TH has been found to induce oxidative stress as well, along with some other effects, such as the decrease in AChE and Na^+^/K^+^-ATPase activity. 

A significant reduction was also found in the monoamine concentrations in TH-exposed embryos. Thiacloprid has been also proved to decrease aspartate, glutamine, glycine, and GABA levels in the brain. These changes may indicate a functional impairment of the nervous system. These altered functions due to TH may be due to the identified oxidative damage and they are probably linked to the neuronal dysfunctions as suggested earlier by Schreck et al. [55]. 

Goyal et al. reported that TH induced neurological disorders in *Gallus domesticus* associated with brain histopathological changes [56]. Similarly, imidacloprid treatment inhibited both AChE and acetylcholine receptors, negatively affected neurogenesis during embryonic development of chicks, reduced cell proliferation, increased cell apoptosis, and altered the brain histoarchitecture [57].

The co-administration of CA and RA with TH induced significant adjustments in the brain neurochemical parameters, in the level of serum amino acids and monoamine neurotransmitters, especially when CA and RA were both inoculated in fertilized eggs. 

The modulatory role of CA on noradrenaline (NA), DA5-HT, accompanied by a significant antioxidant effect, was recorded in mice exposed to chronic restraint stress [58]. Similarly, RA has been reported to prevent the decrease in brain cholinergic markers [59]. Similar findings were reported by Kondo et al., who suggested that RA may normalize the activation of HPA axis, increase the brain content of DA, and improve the depressive status of mice [60]. Rosmarinic acid elevated ACh levels and reduced the brain AChE activity in mice, also improving their memory [61].

The neuroprotective potential of CA and RA was found to be amplificated when they were both administered. The antioxidant activity of both of them may be a property that may be accounted for those effects. 

In fact, TH treatment has been found associated with the alteration of the fear and avoidance behavior of hatchlings, impairments in vestibular and motor functioning, and auditory habituation of chicks. 

The neurobehavioral alterations noted in this study could result from the impact of TH on the CNS, through inhibiting the AChE activity. The latter is an important enzyme involved in the cholinergic neurotransmission and it is vital for muscular functions and normal behaviors. Both suppression of AChE and brain pathological changes could mediate the neurobehavioral abnormalities [62]. 

Thiacloprid exerts its effect possibly by acting both as an antagonist and an agonist on the nicotinic ACh receptors that are involved in neurotoxicosis [63]. It altered the memory and learning activities of honey bees [64]. Similarly, Velisek and Stara reported that exposure of common carp larvae and embryos to TH, negatively affected the hatchlings’ behavior and embryos viability [65]. 

Administration of a maternal single dose of imidacloprid (337 mg/kg) to pregnant rats also resulted in neurobehavioral deficits, with higher expression of the glial fibrillary acidic protein in the hippocampus and in the motor cortex of the offspring [66]. Imidacloprid also decreased the AchE activity, the spontaneous locomotor activity and induced histopathological alterations in the brain cerebellum [62]. Similarly, exposure to low doses of acetamiprid during lactation and gestation periods caused abnormalities in the F1 male mice’s sexual behaviors [67]. 

Additionally, clothianidin poisoning during the same periods showed adverse effects on neurobehavioral indices in F1 mice offspring [68]. 

The present observations suggest a close relationship between these behavioral responses and the decline in the activity of Na^+^/K^+^-ATPase and the levels of neurotransmitters in the exposed embryos, as they elicit important functions in the regulation of behavior. The hypothesis that ROS are the most likely candidates linked to the induction of neuronal alterations was confirmed [3]. 

The present study showed that CA and RA separately or in combination significantly ameliorated the behavioral disorders induced by TH. Considering their effects on AChE and activity and expression of SOD and CAT, the action on behavior may reflect their anti-oxidation potential, other than their modulatory effect on the levels of neurotransmitters and Na^+^/K^+^-ATPase activity. However, their combined use was more effective against TH-induced neurotoxicity. 

The effect of RA on neurobehavioral changes in animals has been previously reported in various studies. Rosmarinic acid (2 mg/kg, i.p.) induced a significant reduction in the immobility duration in the forced swimming task of mice and showed antidepressive-like properties [69]. Low doses of RA (1, 2, or 4 mg/kg) could induce anxiolytic-like action without stimulating DNA damages or altering the locomotor activity in the brain, and inhibit the stress-mediated emotional abnormality [70]. 

Specific studies evidenced that RA protected locomotive ability, cognitive ability, and neuronal cells in the CA1 region of the hippocampus, relieving anxiety behavior in cerebral I/R-injured rats [71]. It could also prevent learning and memory deficits induced by cerebral ischemia [72] and amyloid β (Aβ_25–35_) in mice [61] and by scopolamine in rats [73]. 

Rosmarinic acid also enhanced cognitive performance in the Morris water maze test in rats [74]. Additionally, RA showed potential benefit in preventing the auditory deficits associated with the deficiency of estrogen and administration of D-galactose in Wistar rats by exerting an antioxidant action [75]. It reversed the reduced antioxidant activity, cholinergic alterations, lipid oxidation, and auditory disturbances induced by amyloid β (Aβ), thereby improving the auditory process, suggesting the potential antioxidant role of RA [76]. Moreover, the antioxidant activity of CA may be accounted for its activity in improving the behavioral changes induced by TH and explain its complementary actions with RA. 

**Figure 4:**
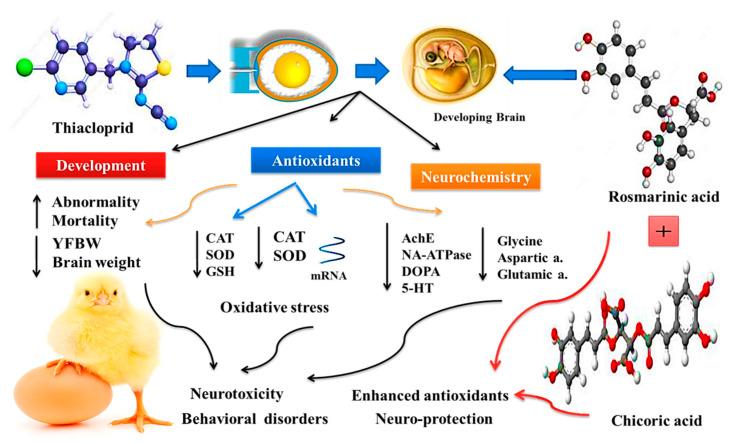
Effect of chicoric (CA) and rosmarinic acids (RA) exposure on suppression of developmental neurotoxicity of thiacloprid (TH) in chicken embryos.

The benefits of using chicoric and rosmarinic acids on mitigating the TH toxicity in the exposed embryos are summarized in Figure 4.

## 5. Conclusions

The current findings suggested that TH-induced developmental neurotoxicity in chicken embryos and altered the brain levels of monoamine neurotransmitters, suppressing the activities of AChE and Na^+^/K^+^-ATPase even at low concentrations. In addition, embryonic exposure to TH influenced the behavioral response of hatchlings indicating that TH could be neurotoxic to embryos of other vertebrates. 

These neurotoxic effects may be associated with oxidative stress and altered expressions of antioxidant related-genes. Chicoric acid and RA, co-administered with TH, markedly attenuated the toxic insult, and provided neuroprotection to the developing brain of TH-intoxicated embryos, where their combined use showed a complementarity. Based on these observations, their use is recommended against neonicotinoids-induced oxidative damage.

## Figures and Tables

**Table 1:** Primer sequences for studied genes used for RT-qPCR.

Gene	Sequences (5′->3′)	Accession Number
SOD1	F: AAAATTACCGGCTTGTCTGATG R: CGCTGGTACACCCATTTG	NM_205064
CAT	F: TGCAAGGCGAAAGTGTTTGA R: CAGATTCTCCAGCAACAGTGGA	NM_001031215
GST-α	F: GGAGAGAGCCTGGATTGATATG R: GGTTGTAGCTCGTTCAGTGAT	NM_001001776
β-actin	F: CCCAAAGCCAACAGAGAGAA R: CCATCACCAGAGTCCATCAC	NM_205518

**Table 2:** Concentration effect of thiacloprid (TH) on the prevalence of malformation, mortality rates, and brain neurochemistry in 19-day-old chicken embryos.

Parameters	Experimental Groups						*p*-Value
	Control	Vehicle	TH_0.1_	TH_1_	TH_10_	TH_100_	
Mortality rate (%)	15.00 ± 0.28 ^d^	15.00 ± 0.028 ^d^	15.18 ± 0.39 ^d^	20.00 ± 0.28 ^c^	23.33 ± 0.01 ^b^	39.99 ± 0.96 ^a^	<0.001
Abnormality rate (%)	0.00 ± 0.00 ^d^	0.00 ± 0.00 ^d^	0.00 ± 0.00 ^d^	10.50 ± 0.57 ^c^	21.34 ± 0.09 ^b^	30.50 ± 1.21 ^a^	<0.001
YFBW ^1^	39.12 ± 0.05 ^a^	38.36 ± 0.02 ^a^	38.30 ± 0.01 ^a^	27.56 ± 0.01 ^b^	25.23 ± 0.01 ^c^	22.45 ± 0.09 ^d^	<0.001
Relative brain weight (g)	0.55 ± 0.01 ^a^	0.55 ± 0.00 ^a^	0.54 ± 0.00 ^a^	0.42 ± 0.00 ^b^	0.34 ± 0.0.02 ^c^	0.30 ± 0.00 ^c^	<0.001
AchE (ng/mg protein)	2.64 ± 0.04 ^a^	2.58 ± 0.02 ^ab^	2.47 ± 0.03 ^b^	1.53 ± 0.02 ^c^	1.40 ± 0.07 ^c^	0.99 ± 0.01 ^d^	<0.001
Dopamine (ng/g)	13.35 ± 0.07 ^a^	13.31 ± 0.14 ^a^	13.21 ± 0.06 ^a^	9.11 ± 0.00 ^b^	8.5 ± 0.12 ^c^	6.18 ± 0.03 ^d^	<0.001
5-HT (ng/g) ^1^	50.57 ± 0.06 ^a^	50.57 ± 0.09 ^a^	50.35 ± 0.02 ^a^	42.67 ± 0.84 ^b^	34.43 ± 3.33 ^c^	19.91 ± 2.22 ^d^	<0.001

Values are mean ± SE, values are not sharing a common superscript letter (a–d) differ significantly at *p* < 0.05. ^1^ Yolk-free body weight (YFBW), serotonin (5-HT).

**Table 3:** Effect of thiacloprid (TH) and/or chicoric (CA) and rosmarinic acids (RA) in ovo exposure on antioxidants and neurochemical parameters of 19-day-old chicken embryos.

Parameters	Experimental Groups							*p*-Value
	Control	TH	CA	RA	TH/CA	TH/RA	TH/CA+RA	
Antioxidants markers								
SOD (U/g tissue)	7.85 ± 0.20 ^a^	3.59 ± 0.30 ^c^	7.97 ± 0.22 ^a^	7.99 ± 0.12 ^a^	5.81 ± 0.06 ^b^	6.47 ± 0.13 ^b^	7.40 ± 0.29 ^a^	<0.001
CAT (U/g tissue)	5.59 ± 0.22 ^ab^	3.13 ± 0.07 ^d^	5.56 ± 0.19 ^ab^	6.09 ± 0.03 ^a^	4.80 ± 0.21 ^c^	5.04 ± 0.20 ^bc^	5.17 ± 0.03 ^bc^	<0.001
GSH (nmol/g tissue)	17.10 ± 0.05 ^a^	13.84 ± 0.91 ^b^	17.18 ± 0.04 ^a^	17.24 ± 0.00 ^a^	16.56 ± 0.32 ^a^	17.07 ± 0.28 ^a^	17.0 ± 0.00 ^a^	<0.001
AchE and monoamines neurotransmitters								
AchE (ng/mg protein)	2.61 ± 0.00 ^b^	1.70 ± 0.05 ^f^	2.70 ± 0.00 ^b^	2.81 ± 0.00 ^a^	1.90 ± 0.00 ^e^	2.10 ± 0.00 ^d^	2.45 ± 0.01 ^c^	<0.001
NA-ATPase (ng/mg protein)	25.39 ± 0.29 ^a^	16.19 ± 0.00 ^c^	26.21 ± 0.00 ^a^	26.53 ± 0.56 ^a^	20.49 ± 1.14 ^b^	21.24 ± 0.57 ^b^	24.02 ± 0.57 ^a^	<0.001
Serotonin (ng/g)	51.49 ± 0.01 ^b^	42.70 ± 0.40 ^d^	55.15 ± 0.00 ^a^	55.87 ± 0.01 ^a^	46.02 ± 0.58 ^c^	48.54 ± 0.59 ^bc^	51.36 ± 1.76 ^b^	<0.001
Dopamine (ng/g)	13.50 ± 0.03 ^a^	10.22 ± 0.00 ^e^	13.66 ± 0.00 ^a^	13.96 ± 019 ^a^	11.00 ± 0.15 ^d^	12.22 ± 0.15 ^c^	12.98 ± 0.49 ^b^	<0.001
GABA (ng/g)	120.03 ± 0.00 ^b^	85.68 ± 0.00 ^d^	122.34 ± 0.02 ^b^	135.82 ± 7.19 ^a^	99.44 ± 0.26 ^c^	100.36 ± 0.34 ^c^	100.93 ± 0.85 ^c^	<0.001
Amino acids neurotransmitters								
Glycine (nmol/mg protein)	2.60 ± 0.00 ^a^	1.26 ± 0.00 ^b^	2.70 ± 0.60 ^a^	2.82 ± 0.22 ^a^	1.90 ± 0.34 ^ab^	2.20 ± 0.17 ^ab^	2.36 ± 0.01 ^ab^	<0.05
Aspartic acid (nmol/mg protein)	3.10 ± 0.00 ^a^	1.18 ± 0.00 ^b^	3.15 ± 0.57 ^a^	3.56 ± 0.00 ^a^	2.43 ± 0.01 ^a^	2.49 ± 0.01 ^a^	2.91 ± 0.28 ^a^	<0.001
Glutamic acid (nmol/mg protein)	0.62 ± 0.01 ^a^	0.37 ± 0.00 ^b^	0.62 ± 0.06 ^a^	0.63 ± 0.04 ^a^	0.60 ± 0.03 ^a^	0.61 ± 0.00 ^a^	0.62 ± 0.01 ^a^	<0.001

Values are mean ± SEM, values are not sharing a common superscript letter (a–d) differ significantly at *p* < 0.05.

## Data Availability

The data presented in this study are available on request from the corresponding authors.
